# Effects of Alcalase and Pepsin Hydrolysis on Gel Forming Behavior and Technological Properties of Porcine Skin Gelatin

**DOI:** 10.3390/gels12080657

**Published:** 2026-07-23

**Authors:** Cheon-Hwang Wi, Jun Hwang, Woo-Young Son, Hyun-Wook Kim

**Affiliations:** 1 Division of Animal Bioscience & Integrated Biotechnology, Gyeongsang National University, Jinju-si 52828, Republic of Korea; cjsghkddl1@naver.com (C.-H.W.); hwangjun1116@naver.com (J.H.); sonwy001223@naver.com (W.-Y.S.); 2 Department of GreenBio Science, Gyeongsang National University, Jinju-si 52725, Republic of Korea

**Keywords:** porcine skin gelatin, gel-forming, enzymatic hydrolysis, viscosity, functional properties

## Abstract

The gelation ability of gelatin is useful for improving texture and stabilizing food structure, but excessive gelation may limit its application in liquid and high-moisture food systems. Therefore, this study compared the effects of Alcalase and pepsin hydrolysis on the gel-forming ability, viscosity, and functional properties of porcine skin gelatin. *o*-Phthaldialdehyde (OPA) analysis showed that the Alcalase hydrolysate had a higher free amino group content (0.77 meqv/g protein) than the pepsin hydrolysate (0.23 meqv/g protein), indicating more extensive peptide bond cleavage (*p* < 0.05). The control formed a stable gel structure after 60 min at 25 °C, whereas both hydrolysates showed reduced gel-forming ability. In particular, the Alcalase hydrolysate was found to maintain high fluidity, completely inhibiting gel formation. Furthermore, 17.5% (*w*/*v*) Alcalase hydrolysate solution showed no measurable viscosity. In contrast, 17.5% (*w*/*v*) pepsin hydrolysate solution exhibited a viscosity of 653.88 cP. In contrast to the Alcalase hydrolysate, the pepsin hydrolysate also showed the highest oil absorption capacity (8.81 g/g), emulsion stability index (854.09 min), and a slightly higher in vitro digestibility (29.36%) (*p* < 0.05). These results demonstrate that enzyme-specific hydrolysis can differentially modify the balance between gel suppression and techno-functional properties of porcine skin gelatin.

## 1. Introduction

Gelatin is a collagen-derived biopolymer widely utilized in food systems because of its unique gel-forming, water-binding, and thickening properties. Owing to the characteristics of cold-set gelation, gelatin has been extensively applied in confectionery, dairy, processed meat, and dessert products to improve texture and structural stability [1]. However, the strong gel-forming behavior of intact gelatin may limit its applicability in liquid food systems where viscosity development is desired without excessive rigid gel formation. In products such as sauces, soups, dressings, and liquid nutritional formulations, excessive gelation can negatively affect flow characteristics, dispersion stability, processing efficiency, and overall product consistency [2]. Thus, controlling gel formation while maintaining desirable techno-functional properties is an important challenge for expanding the utilization of gelatin-derived ingredients in liquid food systems.

Although enzymatic hydrolysis may require additional processing costs compared with simple physical or thermal modification, it has been widely applied as an effective strategy for modulating the molecular and functional properties of food proteins through the cleavage of peptide bonds and the generation of low-molecular-weight peptides. In gelatin systems, hydrolysis-induced structural modifications can alter intermolecular interactions associated with gel formation, viscosity development, and interfacial functionality [3,4]. Previous studies have reported that hydrolysis conditions and enzyme specificity strongly influence the molecular characteristics and functional performance of gelatin hydrolysates [5]. Among commercially available proteases, Alcalase and pepsin are particularly suitable for this purpose because they produce distinct hydrolysis patterns. Alcalase generally promotes extensive hydrolysis due to its broad proteolytic specificity, whereas pepsin generates different peptide populations through relatively selective cleavage under acidic conditions [6]. Previous research has also shown that pig skin collagen hydrolysates prepared with pepsin and Alcalase exhibit different structural and functional properties depending on the degree of hydrolysis [7]. These enzyme-dependent differences may subsequently influence techno-functional properties such as gel-forming behavior, viscosity development, and emulsion-related functionality.

In parallel with these technological interests, increasing evidence has demonstrated that gelatin hydrolysates may possess various physiological and bioactive properties, including antioxidant activity, improved digestibility, and peptide-associated biological functionality depending on hydrolysis conditions and peptide composition [5,8]. These findings have contributed to shifting the perception of gelatin from a relatively low-value structural protein source with limited nutritional quality toward a value-added functional protein ingredient. Consequently, previous studies on gelatin hydrolysates have predominantly focused on bioactive peptides, peptide composition, and physiological functionality.

Despite these advances, relatively limited attention has been paid to the technological repurposing of gelatin hydrolysates as protein-derived ingredients with reduced gel-forming behavior for liquid food systems. In particular, systematic understanding of how enzyme-specific hydrolysis influences gel-forming behavior while simultaneously affecting viscosity development, oil-holding capacity, and emulsion stability remains insufficient. This represents an important knowledge gap because these techno-functional properties are critical determinants of ingredient applicability in formulated liquid foods. Furthermore, although extensive hydrolysis is generally expected to suppress gel formation, it remains unclear whether hydrolysates can retain sufficient functional performance to contribute to viscosity development and structural stabilization in liquid systems. Therefore, the objective of this study was to evaluate the technological and functional properties of porcine skin gelatin hydrolysates produced using Alcalase and pepsin, with particular emphasis on reducing gel-forming ability while retaining functional properties relevant to high-moisture food systems.

## 2. Results and Discussion

Enzymatic hydrolysis altered the structural, antioxidant, technological, and digestibility-related properties of porcine skin gelatin in an enzyme-dependent manner. The following sections describe these changes with particular emphasis on the differences between Alcalase- and pepsin-treated hydrolysates.

### 2.1. Gel-Forming Behavior and Hydrolysis Characteristics

The extent of gelatin hydrolysis was first evaluated in relation to gel-forming behavior, free amino group content, and electrophoretic patterns. These analyses were used to clarify how enzyme-specific peptide bond cleavage affected the structural characteristics of porcine skin gelatin.

#### 2.1.1. Qualitative Visual Observation of Apparent Gel Formation

**Figure 1:**
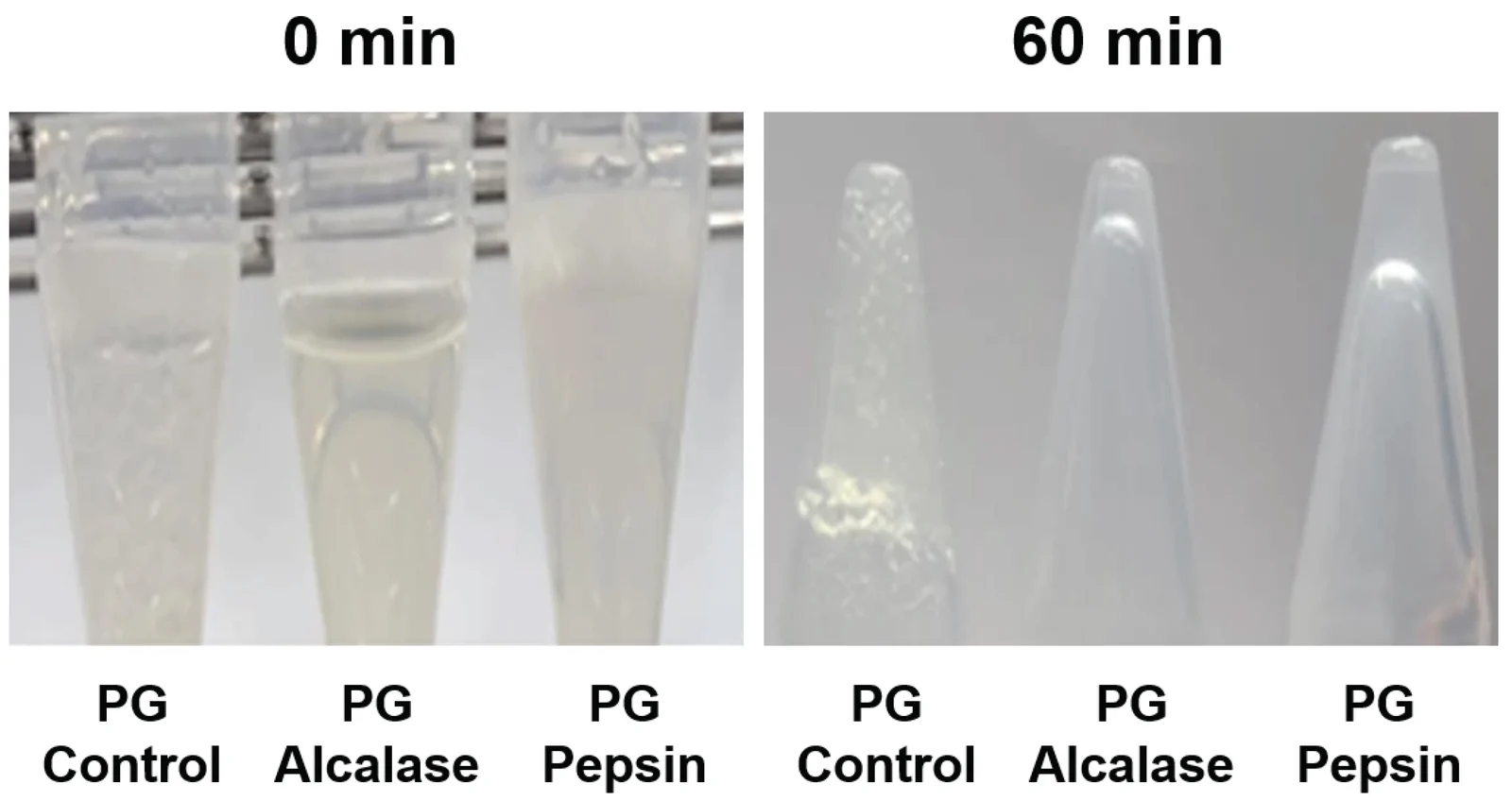
Visual assessment of gel-forming behavior of porcine skin gelatin and its enzymatic hydrolysates during holding at 25 °C. Samples (3%, *w*/*v*) were prepared in deionized distilled water and photographed at 0 and 60 min during holding at 25 °C. PG represents non-hydrolyzed porcine skin gelatin, whereas Alcalase and pepsin represent hydrolysates produced using the respective proteases.

**Figure 2:**
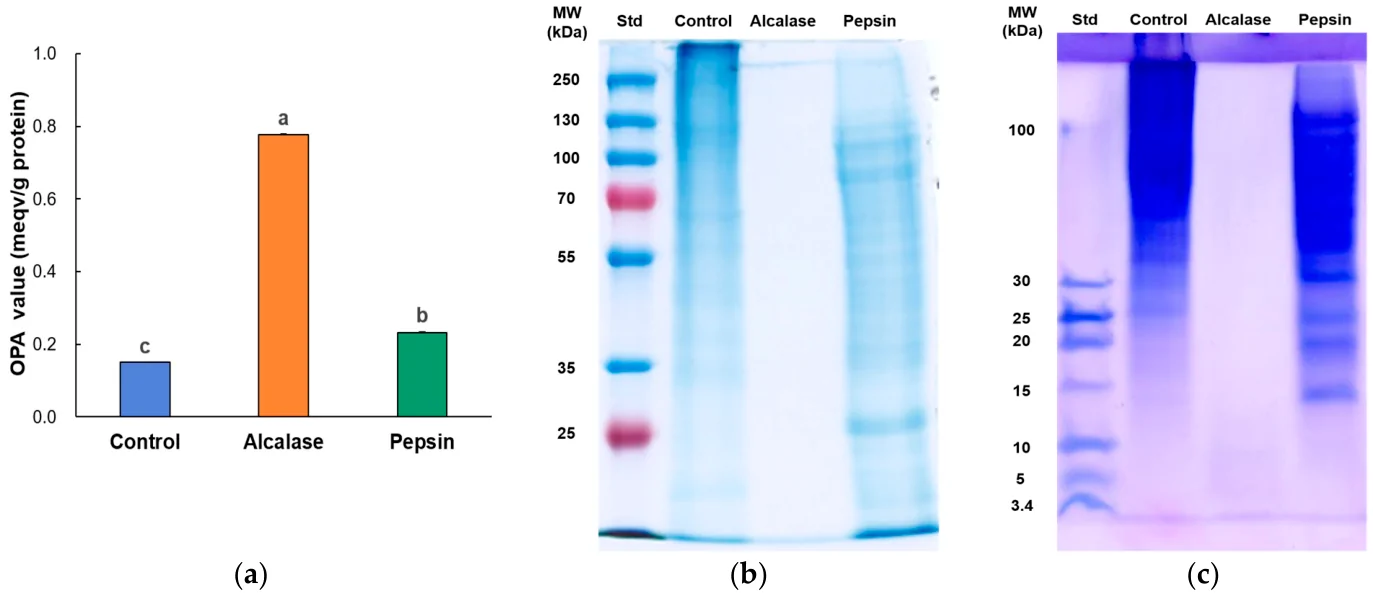
Free amino group content determined by the OPA assay (**a**), representative SDS-PAGE profiles (**b**), and Tricine SDS-PAGE profiles (**c**) of porcine skin gelatin (PG) and its enzymatic hydrolysates produced using Alcalase and pepsin. Free amino group content is expressed as meqv/g protein and presented as mean ± standard deviation (*n* = 3). Different letters in OPA results indicate significant differences among treatments (*p* < 0.05). Std indicates the standard protein marker. Colors are used only to distinguish the bars and do not represent additional variables.

The apparent gel formation of porcine skin gelatin and its enzymatic hydrolysates was qualitatively observed at 25 °C (Figure 1). After 60 min of holding, the control sample showed apparent gel formation, whereas both hydrolysates showed reduced apparent gel formation. The Alcalase hydrolysate remained highly fluid throughout the holding period, suggesting reduced apparent gel formation under the applied conditions, while the pepsin hydrolysate exhibited limited structural stabilization. Gelatin gelation occurs through the reassociation of collagen-derived polypeptide chains during cooling, resulting in the formation of junction zones and a three-dimensional network structure [9]. Thus, extensive peptide bond cleavage during hydrolysis may reduce the ability of gelatin molecules to reassociate into stable gel networks. Similar reductions in gel-forming behavior following enzymatic hydrolysis of gelatin have also been reported previously [10]. Moreover, the greater suppression of gel formation observed in the Alcalase hydrolysate is consistent with its higher OPA-reactive free amino group content and the extensive disappearance of protein bands in the electrophoretic results (Figure 2). In contrast, the pepsin hydrolysate retained a limited ability to form weak structural networks, suggesting that moderate hydrolysis may suppress excessive gelation while preserving some intermolecular interactions. This behavior may be advantageous for high-moisture food systems where flowability is required without the development of rigid gel structures.

#### 2.1.2. Free Amino Group Content of Porcine Skin Gelatin Hydrolysates

Free amino group content was evaluated using the OPA assay as a relative indicator of OPA-reactive primary amino groups generated after enzymatic hydrolysis (Figure 2a). The OPA value of porcine skin gelatin hydrolysates differed depending on the protease used (*p* < 0.05). The Alcalase-treated hydrolysate exhibited the highest OPA value, reaching 0.77 meqv/g protein, whereas the pepsin-treated hydrolysate showed a comparatively lower value of 0.23 meqv/g protein (*p* < 0.05). These results suggest that, under the applied hydrolysis conditions, Alcalase generated a greater amount of OPA-reactive free amino groups than pepsin. This finding indicates that Alcalase generated a greater amount of OPA-reactive free amino groups under the applied hydrolysis conditions than pepsin. However, it should be noted that the OPA assay mainly reacts with primary amino groups, whereas secondary imino groups such as proline and hydroxyproline exhibit relatively low reactivity with OPA [11]. Therefore, considering the glycine-, proline-, and hydroxyproline-rich characteristics of collagen/gelatin substrates, the OPA value should be interpreted as an indicator of OPA-reactive free amino groups rather than a complete representation of all peptide bond cleavage events [12]. In addition, the different cleavage specificities of Alcalase and pepsin may generate different peptide termini and amino acid profiles, which can influence the amount of OPA-reactive amino groups detected by the assay. Previous studies have reported that Alcalase and pepsin exhibit different proteolytic specificities, resulting in the generation of distinct peptide populations from collagen/gelatin substrates [13]. Accordingly, the marked difference in OPA-reactive free amino group content observed in the present study may reflect differences in peptide size distribution, peptide termini, and molecular structure arising from the different cleavage patterns of the two enzymes, which may subsequently influence gel-forming behavior and other techno-functional properties of the resulting hydrolysates.

#### 2.1.3. Electrophoresis Patterns of Porcine Skin Gelatin Hydrolysates

Porcine skin gelatin exhibited markedly different electrophoretic patterns depending on the enzyme treatment applied (Figure 2b,c). In both SDS-PAGE and Tricine SDS-PAGE analyses, distinct protein and peptide bands were observed in the control and pepsin-treated hydrolysate, whereas no visible bands were detected in the Alcalase-treated hydrolysate. These results indicate that Alcalase induced substantially more extensive degradation of gelatin molecules than pepsin under the applied hydrolysis conditions. The disappearance of detectable bands in the Alcalase hydrolysate may be attributed to the generation of low-molecular-weight peptides that were too small and/or too diffuse to be visualized within the electrophoretic systems. In contrast, the pepsin hydrolysate retained visible electrophoretic bands in both SDS-PAGE systems, suggesting that pepsin hydrolysis produced peptide fractions with larger molecular sizes than those generated by Alcalase. This result may be associated with the relatively selective cleavage specificity of pepsin toward peptide bonds adjacent to aromatic amino acid residues, which may limit hydrolysis efficiency in gelatin substrates predominantly composed of glycine-, proline-, and hydroxyproline-rich sequences [14,15]. The retention of detectable peptide bands in the pepsin hydrolysate suggests that moderate hydrolysis preserved peptide fractions capable of contributing to intermolecular interactions, which may be associated with the partial gel-forming behavior and viscosity retention observed in subsequent analyses.

### 2.2. Antioxidant Activities

The antioxidant properties of porcine skin gelatin and its hydrolysates were evaluated using multiple assay systems to account for differences in radical scavenging and reducing mechanisms.

#### 2.2.1. Radical Scavenging Activities

**Figure 3:**
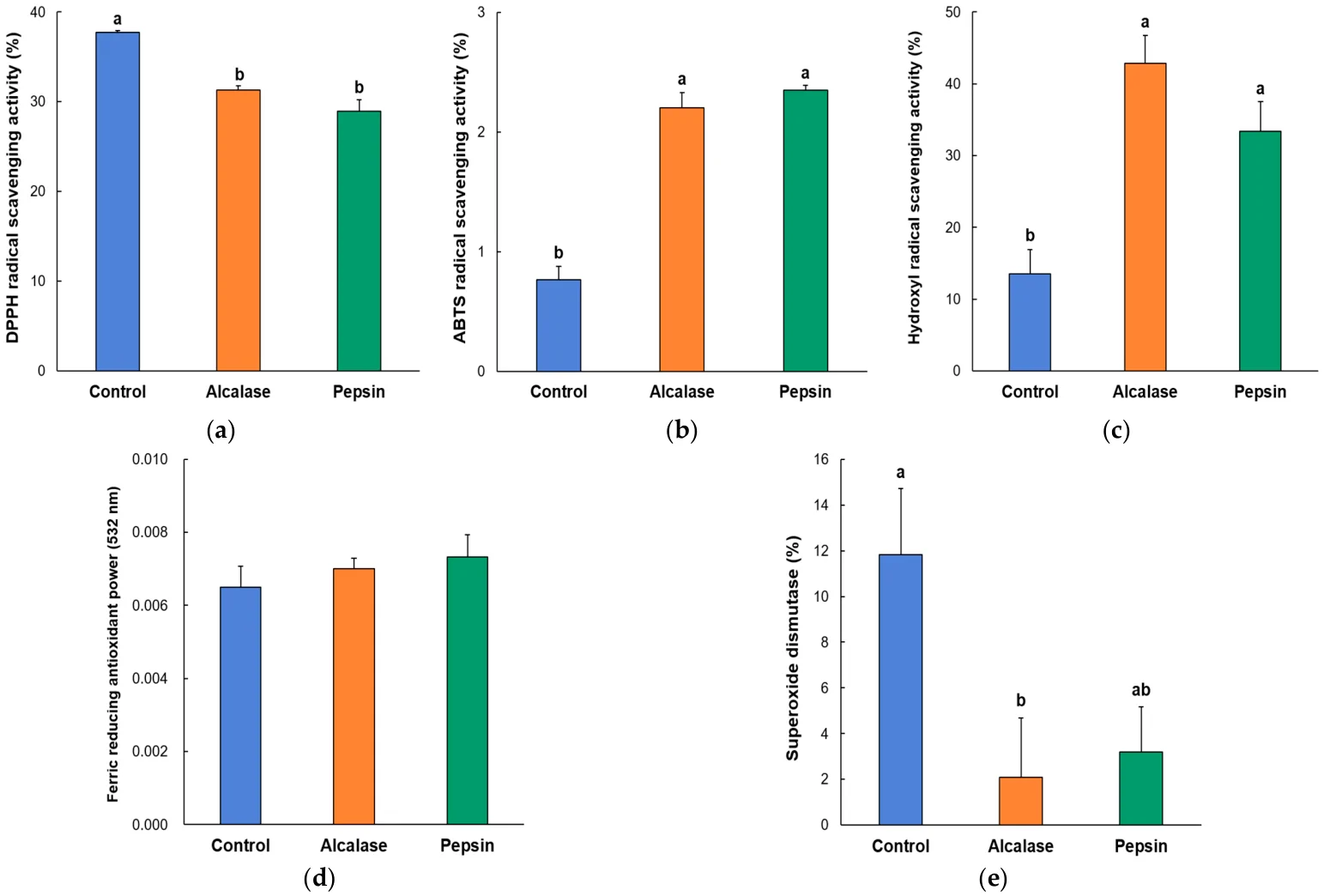
Antioxidant properties of porcine skin gelatin and its hydrolysates produced using Alcalase and pepsin. (**a**) ABTS radical scavenging activity, (**b**) hydroxyl radical scavenging activity, (**c**) DPPH radical scavenging activity, (**d**) ferric reducing antioxidant power (FRAP), and (**e**) superoxide dismutase (SOD)-like activity. PG represents non-hydrolyzed porcine skin gelatin, whereas Alcalase and pepsin represent hydrolysates produced using the respective proteases. Values are expressed as mean ± standard deviation (*n* = 3). Different letters within each panel indicate significant differences among treatments (*p* < 0.05). Values sharing at least one common letter are not significantly different. Colors are used only to distinguish the bars and do not represent additional variables.

Radical scavenging activities of porcine skin gelatin hydrolysates differed depending on both the antioxidant assay system and the protease used for hydrolysis (Figure 3a–c). In the ABTS assay, enzymatic hydrolysis significantly increased radical scavenging activity compared with the non-hydrolyzed control (*p* < 0.05). The control exhibited an activity of 0.77%, whereas higher activities were observed in the Alcalase (2.20%) and pepsin (2.35%) hydrolysates. Similarly, hydroxyl radical scavenging activity increased from 13.54% in the control to 42.83% and 33.38% in the Alcalase and pepsin hydrolysates, respectively. Among the evaluated antioxidant assays, hydroxyl radical scavenging activity exhibited the greatest response to enzymatic hydrolysis. The comparatively higher hydroxyl radical scavenging activity observed in the Alcalase hydrolysate may be associated with its greater degree of hydrolysis, as indicated by the OPA and electrophoretic results (Figure 2). Previous studies have similarly reported that extensive hydrolysis by Alcalase can generate peptide fractions with enhanced radical scavenging activity because of increased exposure of reactive amino acid residues and formation of low molecular weight peptides [13,15].

In contrast, DPPH radical scavenging activity exhibited an opposite response pattern. The control showed the highest DPPH radical scavenging activity (37.75%), whereas lower activities were observed in the Alcalase (31.28%) and pepsin (28.92%) hydrolysates. These results suggest that enzymatic hydrolysis did not uniformly enhance all antioxidant properties of gelatin. The reduced DPPH radical scavenging activity may be associated with hydrolysis-induced changes in peptide size and structure, as previous studies have reported that excessive peptide fragmentation can reduce hydrogen donation and electron transfer capacities in certain antioxidant systems [16]. Overall, the antioxidant responses of porcine skin gelatin hydrolysates were strongly dependent on the assay system employed, indicating that enzymatic hydrolysis altered the antioxidant profile of gelatin rather than consistently enhancing antioxidant activity.

#### 2.2.2. Ferric Reducing Antioxidant Potential (FRAP) of Porcine Skin Gelatin Hydrolysates

Ferric reducing antioxidant power (FRAP) values of porcine skin gelatin and its hydrolysates are presented in Figure 3d. Although the hydrolysates exhibited slightly higher FRAP values than the control, no significant differences were observed among treatments (*p* > 0.05). These results indicate that ferric ion reducing capacity was only marginally influenced by enzymatic hydrolysis under the applied conditions. Similar observations have been reported in gelatin hydrolysates, where reducing power was affected to a limited extent by peptide composition and amino acid distribution [5].

#### 2.2.3. Superoxide Dismutase (SOD)-like Activity of Porcine Skin Gelatin Hydrolysates

Superoxide dismutase (SOD)-like activity of porcine skin gelatin and its hydrolysates is presented in Figure 3e. The control exhibited the highest SOD-like activity, whereas lower activities were observed in both Alcalase and pepsin hydrolysates. In particular, the Alcalase hydrolysate showed the lowest activity among the treatments. These results suggest that enzymatic hydrolysis did not improve SOD-like activity under the applied conditions. The reduction in SOD-like activity may be associated with hydrolysis-induced alterations in peptide structures involved in superoxide radical scavenging. Previous studies have similarly reported that antioxidant responses of protein hydrolysates vary depending on hydrolysis pattern, peptide size distribution, and assay mechanism [17,18]. The lower SOD-like activities observed in the hydrolysates suggest that peptide structures contributing to superoxide radical scavenging were partially disrupted during enzymatic hydrolysis, particularly under the extensive hydrolysis conditions produced by Alcalase.

### 2.3. Technological Properties of Porcine Skin Gelatin Hydrolysates

The technological properties of porcine skin gelatin and its hydrolysates were assessed to determine their potential applicability as protein-derived ingredients in high-moisture food systems.

#### 2.3.1. Water-Holding Capacity and Oil Absorption Capacities of Porcine Skin Gelatin Hydrolysates

**Figure 4:**
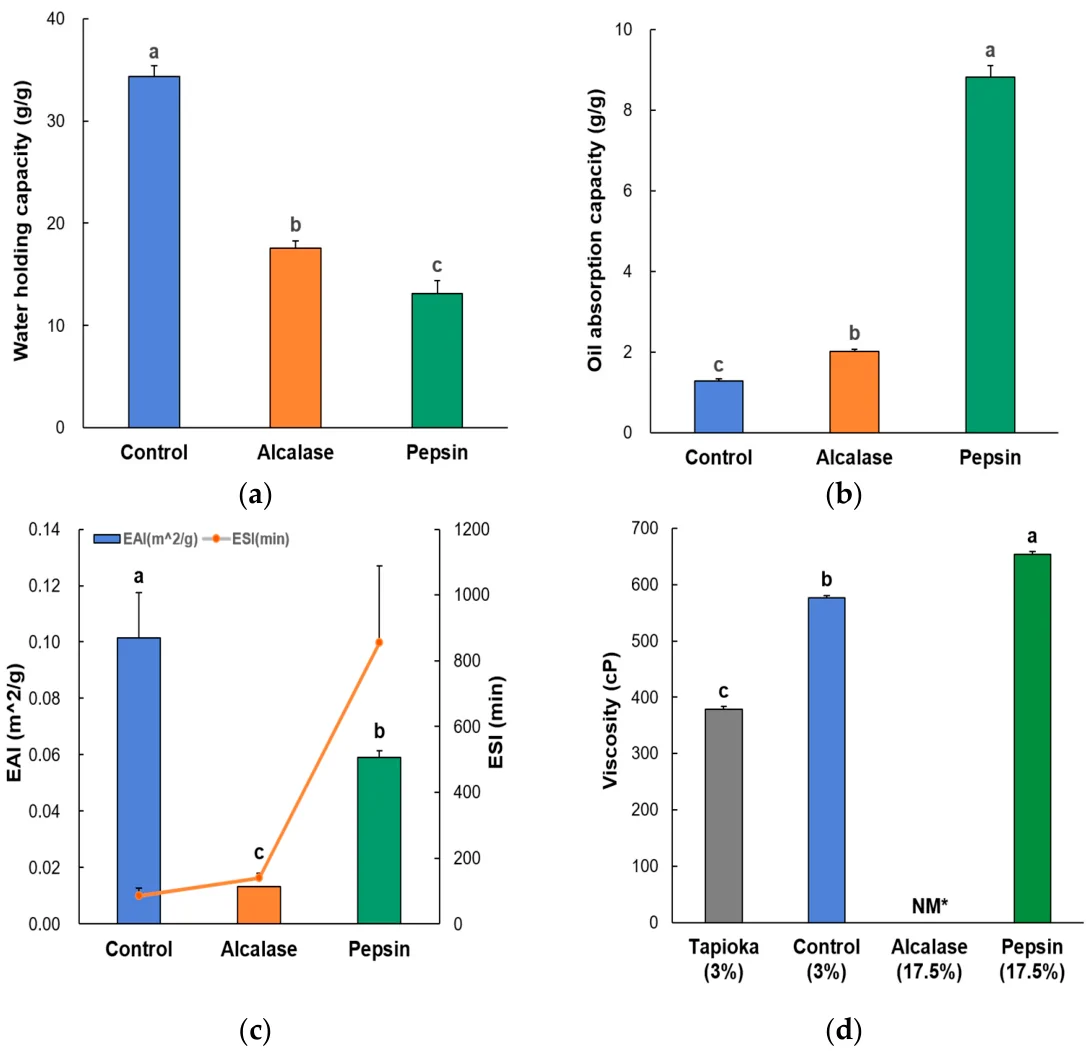
Technological properties of porcine skin gelatin and its hydrolysates produced using Alcalase and pepsin. (**a**) Water-holding capacity (WHC). (**b**) Oil-absorption capacity (OAC). (**c**) Emulsifying activity index (EAI) and emulsion stability index (ESI). (**d**) Viscosity of tapioca starch, porcine skin gelatin, and gelatin hydrolysates. PG represents non-hydrolyzed porcine skin gelatin, whereas Alcalase and pepsin indicate hydrolysates produced using the respective proteases. Values are expressed as mean ± standard deviation (*n* = 3). a–c Different letters indicate significant differences among treatments (*p* < 0.05). NM indicates values below the measurable range of the viscometer. Colors are used only to distinguish the bars and do not represent additional variables.

Water-holding capacity (WHC) and oil-absorption capacity (OAC) of porcine skin gelatin and its hydrolysates are presented in Figure 4a and b, respectively. The control exhibited a WHC value of 0.24 g/g, whereas the Alcalase and pepsin hydrolysates showed lower values of 0.11 and 0.12 g/g, respectively (*p* < 0.05). The lower WHC values of the hydrolysates may be attributed not only to increased solubility following enzymatic hydrolysis but also to enzyme-dependent disruption of the gelatin network and reduction in peptide chain length [19]. Enzymatic hydrolysis disrupts the three-dimensional gelatin network and produces smaller peptides with improved dispersibility in aqueous systems, thereby reducing the ability of the hydrolysates to physically retain water during centrifugation [20]. Thus, the apparent decrease in WHC does not necessarily indicate reduced water affinity, but may reflect the increased solubilization of gelatin-derived peptide fractions.

In contrast, substantial differences were observed for OAC. The control exhibited an OHC value of 1.29 g/g, whereas significantly higher values were observed in the Alcalase (2.02 g/g) and pepsin (8.81 g/g) hydrolysates. Among the treatments, the pepsin hydrolysate exhibited the highest OAC value. These results suggest that enzymatic hydrolysis influenced the oil-binding properties of porcine skin gelatin through enzyme-dependent changes in peptide size, molecular flexibility, and the exposure of hydrophobic amino acid residues capable of interacting with lipid phases [21]. The markedly higher OAC observed -in the pepsin hydrolysate suggests that moderate hydrolysis may favor the formation of peptide structures with balanced hydrophobicity and sufficient molecular size for lipid interaction, whereas extensive hydrolysis by Alcalase may generate excessively small peptides with comparatively lower oil-retention capacity. These results indicate that enzyme-specific hydrolysis patterns can substantially influence lipid interaction properties and potential ingredient functionality in liquid food systems.

#### 2.3.2. Emulsifying Capacity of Porcine Skin Gelatin Hydrolysates

Emulsifying properties of porcine skin gelatin and its hydrolysates are presented in Figure 4c. The control exhibited the highest emulsifying activity index (EAI) value (0.025 m^2^/g), whereas lower EAI values were observed in the Alcalase and pepsin hydrolysates (*p* < 0.05). Among the hydrolysates, the Alcalase treatment showed the lowest EAI value. These results suggest that enzymatic hydrolysis reduced the initial emulsifying activity of gelatin. Similar reductions in EAI following extensive hydrolysis have previously been reported in gelatin and protein hydrolysates, where excessive peptide bond cleavage reduced molecular size and limited the ability of peptides to rapidly adsorb at newly formed oil–water interfaces [22].

In contrast, emulsion stability index (ESI) showed an opposite trend. The control exhibited the lowest ESI value, whereas higher emulsion stability was observed in the hydrolysates, particularly in the pepsin-treated sample (*p* < 0.05). The pepsin hydrolysate exhibited the highest ESI value, indicating improved emulsion stability compared with the control and Alcalase hydrolysate. These results suggest that moderate hydrolysis may favor formation of peptides with suitable molecular size and balanced hydrophobicity capable of forming more stable interfacial layers. Similar enzyme-dependent differences in emulsifying properties have also been reported previously, where moderate hydrolysis improved emulsion stability whereas extensive hydrolysis reduced interfacial functionality because of excessive peptide fragmentation [22].

Overall, the results indicate that enzyme-specific hydrolysis patterns differently influenced emulsifying activity and emulsion stability of porcine skin gelatin hydrolysates. Extensive hydrolysis by Alcalase reduced EAI and did not effectively improve ESI, whereas the pepsin hydrolysate retained comparatively greater emulsion stability despite reduced initial emulsifying activity.

#### 2.3.3. Apparent Viscosity of Porcine Skin Gelatin Hydrolysates

The apparent viscosity values of porcine skin gelatin and its hydrolysates are presented in Figure 4d. The 3% tapioca starch solution exhibited a viscosity of 378.88 cP, whereas the 3% porcine skin gelatin solution showed a higher viscosity of 576.94 cP. In contrast, the Alcalase hydrolysate showed viscosity values below the measurable range of the viscometer, even at 17.5% (*w*/*v*), indicating a substantial loss of viscosity-developing ability. This result is consistent with previous studies reporting that enzymatic hydrolysis, including Alcalase treatment, reduces the molecular weight of gelatin and consequently decreases viscosity- and gelation-related properties [23,24]. Thus, the absence of measurable viscosity in the Alcalase hydrolysate may be attributed to extensive peptide bond cleavage and the formation of low-molecular-weight peptides, which weakened intermolecular interactions.

Conversely, the pepsin hydrolysate exhibited a measurable viscosity of 653.88 cP despite its reduced gel-forming behavior. This suggests that pepsin hydrolysis suppressed rigid gel network formation while partially preserving peptide fractions involved in viscosity development. Supporting this interpretation, the pepsin hydrolysate showed lower free amino group content than the Alcalase hydrolysate and retained detectable peptide bands in both SDS-PAGE and Tricine SDS-PAGE (Figure 2). Previous studies have also reported that pepsin-involved enzymatic hydrolysis alters the electrophoretic pattern and peptide profile of gelatin, and that enzyme type influences the peptide fractions and functional properties of gelatin hydrolysates [25]. Thus, the maintained viscosity of the pepsin hydrolysate is thought to be related to the conservation of a partially conserved peptide chain capable of supporting intermolecular interactions.

It should be noted that the hydrolysates required a substantially higher concentration than native gelatin to achieve measurable viscosity values; therefore, direct comparison of viscosity values among treatments should be interpreted with caution. Nevertheless, these results clearly demonstrate enzyme-dependent differences in viscosity retention following gelatin hydrolysis. Overall, enzyme-dependent differences in measurable viscosity were observed following hydrolysis.

### 2.4. Apparent Digestibility of Porcine Skin Gelatin Hydrolysates

In vitro digestibility of porcine skin gelatin and its hydrolysates is presented in Table 1. The control exhibited an in vitro digestibility of 26.82%, whereas the Alcalase hydrolysate showed a similar value of 26.86% without significant difference from the control (*p* > 0.05). In contrast, the pepsin hydrolysate exhibited significantly higher digestibility (29.36%) than both the control and Alcalase hydrolysate. These results indicate that enzyme-specific hydrolysis patterns influenced susceptibility of gelatin-derived peptides to gastrointestinal digestion. The relatively higher digestibility observed in the pepsin hydrolysate may be associated with the formation of peptide structures more accessible to digestive enzymes during simulated gastrointestinal digestion. Previous studies have similarly reported that enzymatic hydrolysis can modify peptide size distribution and structural characteristics, thereby influencing the digestibility of protein hydrolysates [26]. Although the pepsin hydrolysate showed statistically higher in vitro digestibility than the control and Alcalase hydrolysate, the absolute differences among treatments were relatively small and should therefore be interpreted cautiously. Nevertheless, the results suggest that moderate hydrolysis by pepsin may partially improve the enzymatic accessibility of gelatin-derived peptides while retaining several desirable techno-functional properties.

## 3. Conclusions

Enzymatic hydrolysis significantly altered the structural and techno-functional properties of porcine skin gelatin in an enzyme-dependent manner. Alcalase treatment resulted in higher OPA-reactive free amino group content and stronger suppression of apparent gel formation and almost completely suppressed gel formation, whereas pepsin treatment provided a more favorable balance between reduced gel-forming behavior and retention of viscosity, oil-absorption capacity, emulsion stability, and in vitro digestibility. Although antioxidant responses varied depending on the assay system, the overall results suggest that controlled enzymatic hydrolysis, particularly with pepsin, may be an effective strategy for tailoring the structural and techno-functional properties of gelatin hydrolysates.

From an application perspective, the pepsin hydrolysate may be more suitable than the Alcalase hydrolysate for high-moisture food systems requiring reduced gel formation while retaining viscosity, oil-absorption capacity, and emulsion stability. However, a limitation of this study is that the enzyme activities of the commercial Alcalase and pepsin preparations were not independently verified before hydrolysis. Therefore, the results should be interpreted based on the manufacturer-declared enzyme activities and the applied hydrolysis conditions. In addition, this study was limited to model systems and did not evaluate the performance of gelatin hydrolysates in actual food matrices or under storage conditions. Further studies are required to evaluate the thermal and molecular structural changes in gelatin hydrolysates using differential scanning calorimetry (DSC), thermogravimetric analysis (TG), X-ray powder diffraction (XRPD), and Fourier-transform infrared spectroscopy (FTIR), as well as their performance in actual food matrices and storage conditions.

## 4. Materials and Methods

### 4.1. Preparation of Gelatin Hydrolysates

The enzymatic hydrolysis procedure was performed according to Verma et al. [27], with modifications to the substrate type, enzyme-specific reaction conditions, and post-hydrolysis processing. Commercial porcine skin gelatin (Type A, Bloom strength 220, Geltech Co., Ltd., Busan, Republic of Korea), a collagen-derived protein product composed mainly of polypeptides obtained by partial hydrolysis of porcine skin collagen, was dissolved in deionized-distilled water (DDW) at a concentration of 5% (*w*/*v*) by heating at 60 °C for 15 min for hydrolysate preparation. This concentration was selected because it allowed complete dissolution of gelatin under the applied heating conditions while providing a sufficient amount of freeze-dried hydrolysate powder for subsequent analyses. After complete dissolution, the solution pH was adjusted to either 8.00 or 2.00 using 0.1 M NaOH or 1 N HCl, respectively, prior to enzymatic hydrolysis. Alcalase 2.4L (EC 3.4.21.62; activity 2.4 AU/kg; density 1.18 g/mL; Novozymes, Bagsværd, Denmark) was added to the gelatin solution adjusted to pH 8.00, whereas pepsin (EC 3.4.23.1; ≥250 units/mg solid; Sigma-Aldrich, St. Louis, MO, USA) was added to the solution adjusted to pH 2.00. The enzyme activities used in this study were based on the manufacturer’s declared specifications, and additional experimental verification of enzyme activity was not performed before hydrolysis. The enzyme-to-substrate ratio (E:S) was set at 1% (*w*/*w*) based on protein content for both enzymes. Hydrolysis conditions for each protease were selected based on previously reported optimal reaction conditions for each enzyme [27]. Enzymatic hydrolysis was conducted for 2 h in a thermostatically controlled water bath (JSIB-22T, JS Research Inc., Gongju-si, Republic of Korea) at 50 °C for Alcalase and 37 °C for pepsin. During hydrolysis, the reaction mixtures were continuously stirred using a magnetic stirrer (HS-50A, DAIHAN Scientific Co., Ltd., Wonju-si, Republic of Korea). Following hydrolysis, enzymatic reactions were terminated by heating the mixtures at 85 °C for 20 min. The hydrolysates were subsequently cooled to room temperature and filtered through a stainless-steel test sieve (500 µm mesh size; model 885705; Chung Gye Sang Gong Sa, Seoul, Republic of Korea) to remove undissolved aggregates and insoluble residues. The filtered hydrolysates were freeze-dried using a laboratory freezer (80 × 10^−3^ Torr pressure, PVTFD10R, Ilshin Lab Co., Daejeon-si, Republic of Korea) and pulverized into powder form prior to analysis. The resulting powders were stored at 4 °C in airtight containers and used for subsequent analyses within 24 h after reconstitution.

### 4.2. Structural Characterization and Gel-Forming Behavior

The structural characteristics of porcine skin gelatin and its hydrolysates were evaluated through visual gel formation, free amino group analysis, and electrophoretic profiling.

#### 4.2.1. Visual Gel Formation

The gel-forming behavior of porcine skin gelatin and its enzymatic hydrolysates was qualitatively evaluated by visual observation during storage at 25 °C. For the visual gel-forming assay, porcine skin gelatin and its hydrolysates were reconstituted in deionized-distilled water (DDW) at a concentration of 3% (*w*/*v*), which was selected to clearly compare gel formation and flowability among treatments under the same evaluation conditions. Then, 2 mL aliquots of each sample solution were transferred into 15 mL conical tubes. The samples were heated at 60 °C for 15 min to ensure complete dissolution and subsequently maintained at 25 °C. Photographs were obtained at 0 and 60 min during holding at 25 °C. Gel formation was qualitatively assessed based on visual changes in structural integrity and apparent gelation behavior.

#### 4.2.2. Free Amino Group Content

The free amino group content of porcine skin gelatin and its enzymatic hydrolysates was determined using the *o*-phthaldialdehyde (OPA) method described by Nielsen et al. [28], with minor modifications. Standard, blank, and sample solutions were prepared in quadruplicate for each treatment. Briefly, 3 mL of freshly prepared OPA reagent containing 3.81% (*w*/*v*) sodium tetraborate decahydrate, 0.1% (*w*/*v*) sodium dodecyl sulfate (SDS), 0.08% (*w*/*v*) OPA dissolved in 4 mL ethanol, and 0.09% (*w*/*v*) dithiothreitol (DTT) was added to each test tube. Subsequently, 400 µL of _L_-serine standard solution was added to the standard tubes, whereas 400 µL of gelatin or gelatin hydrolysate solution was added to the sample tubes. DDW (400 µL) was added to the blank tubes. The mixtures were gently vortexed and allowed to react at room temperature for 2 min. Absorbance was measured at 340 nm using a spectrophotometer, and the reagent blank was used as the reference. The serine standard was analyzed four times (twice before and twice after sample analysis), and the average absorbance value was used for calculation. Results were expressed as serine-NH_2_ equivalents (meqv/g protein) according to Equation (1):

(1) serine-NH_2_ = {(OD_sample_ − OD_blank_)/(OD_standard_ − OD_blank_)} × (0.9516 meqv/L × 0.1 × 100)/(X × P).

where OD_sample_ is the absorbance of the sample, OD_standard_ is the absorbance of the serine standard, OD_blank_ is the absorbance of the blank, X is the sample weight in grams, and P is the protein content of the sample.

#### 4.2.3. Protein Electrophoresis

The protein molecular weight distribution of porcine skin gelatin and its enzymatic hydrolysates was analyzed using SDS-PAGE and Tricine SDS-PAGE according to the methods of Laemmli [29] and Schägger [30], respectively, with minor modifications. For SDS-PAGE, samples were diluted to a final protein concentration of 20 mg/mL and mixed with sample buffer containing 60 mM Tris-HCl (pH 6.8), 25% (*v*/*v*) glycerol, 2% (*w*/*v*) SDS, 14.1 mM β-mercaptoethanol, and 0.1% (*w*/*v*) bromophenol blue at a ratio of 4:1 (sample:buffer). For Tricine SDS-PAGE, samples were diluted to 5 mg/mL and mixed with sample buffer containing 4% (*w*/*v*) SDS, 12% (*v*/*v*) glycerol, 50 mM Tris-HCl, 2% (*v*/*v*) β-mercaptoethanol, and 0.01% (*w*/*v*) Serva Blue G (pH 6.8) at a ratio of 4:1 (*v*/*v*). All sample mixtures were heat-denatured at 100 °C for 5 min prior to electrophoresis. Electrophoresis was conducted using a Mini-PROTEAN Tetra Cell system (Bio-Rad, Hercules, CA, USA). For SDS-PAGE, samples were separated using 5% stacking and 12% resolving gels at 80 V and 120 V, respectively. A molecular weight marker (Precision Plus Protein™ Dual Color Standards, Bio-Rad, Hercules, CA, USA) was used for protein size estimation. For Tricine SDS-PAGE, electrophoresis was performed using Tris-Tricine buffer systems at 30 V for stacking and 150 V for separation. A low-molecular-weight protein marker (PageRule™ Low Range Unstained Protein Ladder; Thermo Scientific, Vilnius, Lithuania) was used for peptide molecular weight estimation. After electrophoresis, gels were stained with Coomassie Brilliant Blue R-250 or Serva Blue G staining solutions and subsequently destained with acetic acid-containing solutions until clear visualization of protein and peptide bands was achieved.

### 4.3. Antioxidant Activity

#### 4.3.1. ABTS Radical Scavenging Activity

The ABTS radical scavenging activity of porcine skin gelatin hydrolysates was determined according to the method described by Cui et al. [31], with minor modifications. ABTS radical cation (ABTS^·+^) solution was prepared by reacting 7 mM ABTS with 2.45 mM potassium persulfate and incubating the mixture in the dark at room temperature for 16 h. Prior to analysis, the ABTS^·+^ solution was diluted with distilled water to obtain an absorbance of 0.70 ± 0.02 at 734 nm. Subsequently, 40 µL of sample solution (1 mg/mL) was mixed with 5 mL of diluted ABTS^·+^ solution and incubated in the dark at room temperature for 6 min. Absorbance was measured at 734 nm using a spectrophotometer. ABTS radical scavenging activity was calculated as Equation (2):

(2) ABTS radical scavenging activity (%) = (A_blank_ − A_sample_)/A_blank_ × 100.

where A_blank_ is the absorbance of the blank solution prepared using distilled water instead of the sample, and A_sample_ is the absorbance of the sample solution.

#### 4.3.2. DPPH Radical Scavenging Activity

DPPH radical scavenging activity was determined according to the method described by Cui et al. [31], with minor modifications. Briefly, sample solution (1 mg/mL) was mixed with 0.1 mM DPPH solution prepared in ethanol and incubated at 25 °C for 30 min in the dark. Absorbance was subsequently measured at 515 nm using a spectrophotometer. DPPH radical scavenging activity was calculated using Equation (3):

(3) DPPH radical scavenging activity (%) = [1 − (A_sample_ − A_blank_)/A_control_] × 100.

where A_blank_ is the absorbance of the blank solution prepared by mixing the sample with ethanol at a 1:1 ratio, A_sample_ is the absorbance of the reaction mixture prepared by mixing the sample with DPPH solution at a 1:1 ratio, and A_control_ is the absorbance of the control solution prepared by mixing DPPH solution with distilled water at a 1:1 ratio.

#### 4.3.3. Hydroxyl Radical Scavenging Activity

Hydroxyl radical scavenging activity was evaluated according to the method described by Chen et al. [32], with minor modifications. Reaction mixtures containing 1,10-phenanthroline, FeSO_4_, phosphate buffer, and sample solution (1 mg/mL) were incubated with hydrogen peroxide at 37 °C for 60 min. Absorbance was measured at 515 nm using a microplate reader (Infinite M200 PRO, Tecan, Männedorf, Switzerland). Hydroxyl radical scavenging activity was calculated as Equation (4):

(4) Hydroxyl radical scavenging activity (%) = (A_sample_ − A_control_)/(A_blank_ − A_control_) × 100.

where A_sample_ is the absorbance of the sample reaction mixture, A_control_ is the absorbance of the control solution prepared using distilled water instead of the sample, and A_blank_ is the absorbance of the blank solution containing 1,10-phenanthroline and FeSO_4_.

#### 4.3.4. Ferric Reducing Antioxidant Power (FRAP)

Ferric reducing antioxidant power (FRAP) was determined according to the modified method of Othman et al. [33]. Sample solution (1 mg/mL) was mixed with phosphate buffer and 1% (*w*/*v*) potassium ferricyanide and incubated at 50 °C for 20 min. After reaction termination with 10% (*w*/*v*) trichloroacetic acid, the mixture was reacted with ferric chloride solution and incubated at 37 °C for 10 min. Absorbance was measured at 700 nm using a spectrophotometer. _L_-ascorbic acid was used as a positive control, and reducing power was expressed based on absorbance values.

#### 4.3.5. Superoxide Dismutase (SOD)-like Activity

Superoxide dismutase (SOD)-like activity of porcine skin gelatin hydrolysates was determined using an OxiTec™ SOD Assay Kit (BIMEX Co., Ltd., Seoul, Republic of Korea) according to the manufacturer’s instructions. The WST working solution was prepared by mixing 1 mL of WST solution with 19 mL of buffer solution. The enzyme working solution was prepared by mixing 16.5 µL of xanthine oxidase solution with 2.5 mL of dilution buffer. Sample solutions were diluted to a final concentration of 1 mg/mL prior to analysis. Aliquots (20 µL) of diluted sample solution were added to the sample wells and blank2 wells of a 96-well microplate. Distilled water (20 µL) was added to blank1 and blank3 wells instead of sample solution. Subsequently, 20 µL of enzyme working solution was added to the sample wells and blank1 wells, followed by gentle mixing. The reaction mixtures were incubated at 37 °C for 20–30 min, and absorbance was measured at 450 nm using a spectrophotometer. SOD was calculated as Equation (5):

(5) SOD-like activity (inhibition rate %) = {(OD_blank1_ − OD_blank3_) − (OD_sample_ − OD_blank2_)}/{(OD_blank1_ − OD_blank3_)} × 100.

### 4.4. Technological Properties

#### 4.4.1. Water-Holding Capacity and Oil Absorption Capacity

Water-holding capacity (WHC) and oil absorption capacity (OAC) were determined according to the method described by Acosta-Domínguez et al. [34], with minor modifications. Briefly, 1.0 g of sample was placed in a 15 mL centrifuge tube, followed by the addition of 10 mL of DDW for WHC analysis or 10 mL of commercial soybean oil for OAC analysis. The mixtures were vortexed for approximately 5 s using a vortex mixer (Vortex-Genie 2, Scientific Industries, Bohemia, NY, USA) and then centrifuged at 3000× *g* for 10 min at 4 °C. After centrifugation, the supernatant was discarded, and the retained water or oil content was determined gravimetrically. WHC and OAC were expressed as grams of retained water or oil per gram of sample and calculated using Equations (6) and (7):

(6) WHC (g/g) = (Water holding sample weight (g) − sample weight (g))/sample weight (g)

(7) OAC (g/g) = (Oil absorption sample weight (g) − sample weight (g))/sample weight (g)

#### 4.4.2. Emulsifying Capacity

Emulsifying activity index (EAI) and emulsion stability index (ESI) were determined according to the method of Pearce et al. [35], with minor modifications. Briefly, 10 mL of soybean oil was mixed with 30 mL of sample solution (5 mg/mL in DDW) and homogenized at 12,000 rpm for 3 min using a homogenizer. Subsequently, 50 µL of the emulsion was mixed with 10 mL of 0.1% (*w*/*v*) SDS solution, and absorbance was measured at 500 nm immediately after homogenization (A0) and after 30 min (A30). EAI and ESI were calculated using Equations (8) and (9):

(8) EAI (m^2^/g) = 2 × 2.303 × A0 × 200/10,000 × C × Ø.

(9) ESI (min) = A0/(A0 − A30) × 30.

where C is the protein concentration of the sample solution (g/mL), Ø is the oil volume fraction, and A0 and A30 are the absorbance values measured at 0 and 30 min, respectively.

#### 4.4.3. Apparent Viscosity

The viscosity of porcine skin gelatin hydrolysates and tapioca starch was measured according to the modified methods of Mulyani et al. [36] and Babic et al. [37]. Tapioca starch, porcine skin gelatin, and gelatin hydrolysates were dissolved in distilled water at concentrations of 3% (*w*/*v*), 3% (*w*/*v*), and 17.5% (*w*/*v*), respectively, with a final volume of 100 mL. Preliminary experiments indicated that hydrolysate solutions at concentrations below 17.5% (*w*/*v*) produced torque values below the recommended operating range of the viscometer. Thus, 17.5% (*w*/*v*) was selected as the minimum concentration allowing reliable viscosity measurement. The relatively high concentration of gelatin hydrolysates was selected based on preliminary experiments because lower concentrations produced viscosity values below the measurable range of the instrument. The tapioca starch solution was heated at 95 °C, whereas gelatin and gelatin hydrolysate solutions were heated at 60 °C for 15 min using a shaking water bath (MaXturdy 45, DAIHAN Scientific Co., Ltd., Wonju-si, Republic of Korea) to ensure complete dissolution. After heating, all samples were cooled and equilibrated at 25 °C for 30 min. Viscosity measurements were performed using a rotational viscometer (DV3T, AMETEK Brookfield, Middleboro, MA, USA) equipped with an SC4-21 spindle at 25 °C and a rotational speed of 40 rpm.

### 4.5. Apparent Digestibility

In vitro digestibility of porcine skin gelatin and its enzymatic hydrolysates was evaluated using a two-step static digestion model simulating gastric and small intestinal digestion according to the method of Biagi et al. [38], with minor modifications and based on the general framework proposed by Brodkorb et al. [39]. Briefly, 0.25 g of each sample was suspended in distilled water, and the pH was adjusted to 2.0 using 1 N HCl to simulate gastric conditions. Pepsin was subsequently added, and the mixtures were incubated at 39 °C for 2 h under continuous agitation. After gastric digestion, the pH was adjusted to 6.8 using 1 N NaOH to simulate intestinal conditions. Pancreatin and bile salts were then added, followed by further incubation at 39 °C for 4 h under continuous agitation. The incubation temperature was selected to reflect physiological digestion conditions commonly used for protein digestibility evaluation [38]. After completion of digestion, the digested samples were centrifuged to separate the soluble fraction from the insoluble residue. No filtration step was performed in this assay. The resulting pellet was collected and dried in a dry oven at 65 °C overnight to remove residual moisture. The dried residue was weighed and used for the gravimetric calculation of apparent in vitro digestibility. In vitro digestibility was calculated according to the following Equation (10):

(10) In vitro digestibility (%) = 100 − [(undigested fraction weight (g) × 100)/sample weight (g)].

### 4.6. Statistical Analysis

All experiments were conducted using three independent batches, and results are presented as mean ± standard deviation. No data points were excluded as outliers. A formal normality test was not conducted because of the limited number of independent observations per treatment. Data were analyzed using one-way analysis of variance (ANOVA) with PASW Statistics version 18.0 (SPSS Inc., Chicago, IL, USA). When significant treatment effects were detected (*p* < 0.05), mean values were separated using Duncan’s multiple range test.

## Figures and Tables

**Table 1:** In vitro digestibility of porcine skin gelatin and hydrolysates produced by Alcalase and pepsin treatment.

Control	Alcalase	Pepsin
26.82 ± 0.02 ^b^	26.86 ± 0.13 ^b^	29.36 ± 0.63 ^a^

PG represents non-hydrolyzed porcine skin gelatin, whereas Alcalase and pepsin indicate hydrolysates produced using the respective proteases. Values are expressed as mean ± standard deviation (*n* = 3). ^a,b^ Different letters indicate significant differences among treatments (*p* < 0.05).

## Data Availability

The original contributions presented in this study are included in the article Further inquiries can be directed to the corresponding author.

## Abbreviations

The following abbreviations are used in this manuscript: ABTS, 2,2′-azino-bis(3-ethylbenzothiazoline-6-sulfonic acid); ANOVA, analysis of variance; DDW, deionized-distilled water; DPPH, 2,2-diphenyl-1-picrylhydrazyl; EAI, emulsifying activity index; ESI, emulsion stability index; FRAP, ferric reducing antioxidant power; OAC, oil absorption capacity; OPA, o-phthaldialdehyde; PG, porcine skin gelatin; SDS-PAGE, sodium dodecyl sulfate-polyacrylamide gel electrophoresis; SOD, superoxide dismutase; WHC, water-holding capacity.
